# Utilizing machine-learning techniques on MRI radiomics to identify primary tumors in brain metastases

**DOI:** 10.3389/fneur.2024.1474461

**Published:** 2025-01-06

**Authors:** W. L. Yang, X. R. Su, S. Li, K. Y. Zhao, Q. Yue

**Affiliations:** ^1^Department of Radiology, West China Hospital, Sichuan University, Chengdu, Sichuan, China; ^2^Department of Radiology, Key Laboratory of Birth Defects and Related Diseases of Women and Children, Ministry of Education, West China Second University Hospital, Sichuan University, Chengdu, Sichuan, China; ^3^Department of Radiology, West China Hospital of Medicine, Huaxi MR Research Center (HMRRC), Chengdu, Sichuan, China; ^4^West China Hospital of Sichuan University, Chengdu, Sichuan, China

**Keywords:** radiomics, machine learning, magnetic resonance imaging, brain metastases, support vector machine (SVM), logistic regression (LR)

## Abstract

**Objective:**

To develop a machine learning-based clinical and/or radiomics model for predicting the primary site of brain metastases using multiparametric magnetic resonance imaging (MRI).

**Materials and methods:**

A total of 202 patients (87 males, 115 females) with 439 brain metastases were retrospectively included, divided into training sets (brain metastases of lung cancer [BMLC] *n* = 194, brain metastases of breast cancer [BMBC] *n* = 108, brain metastases of gastrointestinal tumor [BMGiT] *n* = 48) and test sets (BMLC *n* = 50, BMBC *n* = 27, BMGiT *n* = 12). A total of 3,404 quantitative image features were obtained through semi-automatic segmentation from MRI images (T1WI, T2WI, FLAIR, and T1-CE). Intra-class correlation coefficient (ICC) was used to examine segmentation stability between two radiologists. Radiomics features were selected using analysis of variance (ANOVA), recursive feature elimination (RFE), and Kruskal–Wallis test. Three machine learning classifiers were used to build the radiomics model, which was validated using five-fold cross-validation on the training set. A comprehensive model combining radiomics and clinical features was established, and the diagnostic performance was compared by area under the curve (AUC) and evaluated in an independent test set.

**Results:**

The radiomics model differentiated BMGiT from BMLC (13 features, AUC = 0.915 ± 0.071) or BMBC (20 features, AUC = 0.954 ± 0.064) with high accuracy, while the classification between BMLC and BMBC was unsatisfactory (11 features, AUC = 0.729 ± 0.114). However, the combined model incorporating radiomics and clinical features improved the predictive performance, with AUC values of 0.965 for BMLC vs. BMBC, 0.991 for BMLC vs. BMGiT, and 0.935 for BMBC vs. BMGiT.

**Conclusion:**

The machine learning-based radiomics model demonstrates significant potential in distinguishing the primary sites of brain metastases, and may assist screening of primary tumor when brain metastasis is suspected whereas history of primary tumor is absent.

## Highlights

- Machine learning radiomics models can predict the tumor types of brain metastases.
- Quantitative features from multiparametric MR images may serve as biomarkers for tumor classification.
- Combining clinical features with radiomics models can enhance diagnostic performance.

## Introduction

Brain metastasis (BM) is the most common central nervous system tumor in adults. Patients with BM have a poor prognosis and are still associated with lower overall survival rates, with 2-year OS rates of 8.1% and 5-year OS rates of 2.5% for all tumor types after diagnosis (1–3). The exact incidence rate of BM is currently unclear, although some research reports suggest occurrence rates of 9–17% among cancer patients. However, the aging population leads to an increasing number of cancer diagnoses each year, which in turn raises the likelihood of developing BM, further exacerbated by the widespread application of more sensitive imaging technologies (4).

Reportedly, the incidence rates of BM vary significantly depending on the primary cancer site, with lung cancer (LC) at 41–56%, breast cancer (BC) at 13–30%, malignant melanoma (MM) at 6–11%, and gastrointestinal tumors (GI) at 6–9%. Nevertheless, some studies indicated that a certain proportion of patients (2–14%) present with BM as the initial manifestation of an unknown primary tumor (5, 6). Asymptomatic BM are typically only detectable through screening or postmortem examinations (7). Current guidelines from the European Neurological Society and the European Neuro-Oncology Association highlight the clinical challenges faced in the diagnosis and treatment of brain metastasis patients with unknown primary tumors (8). Due to the need for additional imaging modalities (such as chest CT, abdominal CT, and whole-body PET) and invasive biopsies for more precise diagnosis, patients with unclear primary lesions at the time of brain metastasis diagnosis often undergo multiple steps and extensive technical setups (9). For these patients, rapidly and efficiently determining the primary lesion site is crucial for their individual treatment planning.

Radiomics utilizes quantitative features extracted from segmented images, which are difficult or even impossible to be identified through visual inspection, to find associations with clinically relevant outcomes. In the context of metastatic tumors, radiomics has been evaluated for determining the primary tumor types and mutation status, as well as assessing tumor response post-treatment (10–12). We hypothesize that radiomics models from different MR sequence images can help differentiate BM originating from different primary sites. Our study aimed to explore the feasibility of multi-class machine learning for predicting the primary site of BM based on MRI features.

## Materials and methods

### Study participants

Retrospectively collected data from patients with BM at our institution over a five-year period (December 2017 to December 2022). Primary tumors of BM included lung cancer, breast cancer, gastrointestinal tumors, melanoma, renal cancer, reproductive system tumors, and other malignant tumors. Inclusion criteria were as follows: (1) pathologically confirmed LC, BC, or GI, with only one primary tumor; (2) no specific treatment for their BMs (radiotherapy, surgery, chemotherapy, or targeted therapy); (3) all BM confirmed through imaging and clinical follow-up. Exclusion criteria were as follows: (1) metastases were too small (longest diameter < 9 mm), as texture information could not be accurately captured within small areas by radiomics analysis software; (2) no pathological diagnosis results or more than one primary tumor; (3) pathological types were melanoma, renal cancer, or other tumors rather than the aforementioned three kinds of primary tumors; (4) received treatment for BM; (5) incomplete image sequences, poor image quality, low resolution, and significant image artifacts.

The clinical and radiological data of these patients were independently reviewed by two experienced neuroradiologists (with over 10 years of diagnostic experience), who were blinded to the pathological results. In instances of discordance in radiological observations, consensus was achieved through deliberation between the two clinicians, and the outcomes were subsequently documented by a third physician for statistical scrutiny. The imaging features included single/multiple BM, infratentorial and supratentorial distribution of BM, peritumoral edema, enhancement patterns, tumor shape, cystic degeneration/necrosis of BM, and low signal intensity on T2WI. (1) Whether BMs are single or multiple; (2) The infratentorial and supratentorial distribution of BM; (3) Whether there is peritumoral edema in BM; (4) Enhancement patterns are defined as heterogeneous (mixed irregular areas of tumor enhancement), rim-like enhancement (enhancement at the periphery of the tumor), and indistinct enhancement; (5) The shape of BM is defined as the interface between the tumor and normal brain parenchyma, categorized into single nodular, multi-nodular fusion, and fuzzy edge (indistinct) margins; (6) Tumor cystic degeneration/necrosis refers to areas lacking enhancement; and (7) Whether there are areas of low signal intensity on T2-weighted imaging in tumors.

**Figure 1:**
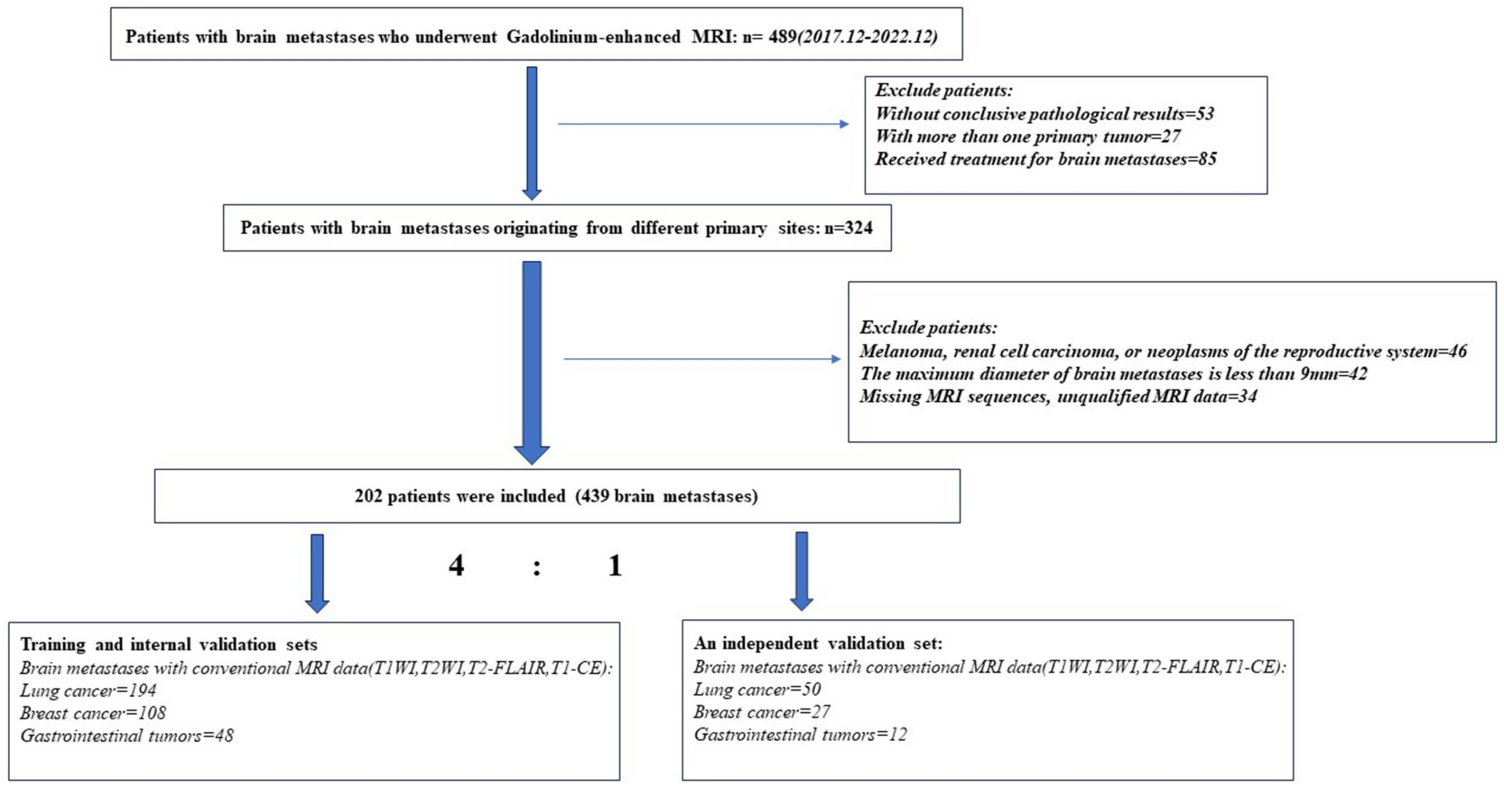
Flowchart of patients’ enrollment process.

A total of 202 patients were finally included (115 females [mean age 61.3 years; range 34–81 years], 87 males [mean age 60.5 years; range 38–86 years]), with a total of 439 brain metastatic lesions (Table 1): 244 originated from LC, 135 from BC, and 60 from GI. The process of patients’ enrollment is shown in Figure 1.

**Table 1:** Baseline demographic and clinical characteristics of all patients.

Characteristic	Lung cancer (*n* = 127, 244^a^)	Breast cancer (*n* = 59, 135^a^)	Gastrointestinal cancer (*n* = 16, 60^a^)	*p* value
Gender				
Male	72 (56.7%)	5 (8.5%)	10 (62.5%)	<0.001
Female	55 (43.3%)	54 (91.5%)	6 (37.5%)	
Age (years old)	63.7 ± 11.3	56.2 ± 10.5	60.4 ± 12.6	0.27
Average metastases size (mm)^a^	14.5 ± 7.8	16.3 ± 8.6	16.8 ± 10.2	0.16
Average metastases volume (cm^3^)^a^	1.55 ± 4.9	2.31 ± 6.4	2.48 ± 6.7	0.08
Number of metastases				
Single	21 (16.5%)	13 (22.0%)	2 (12.5%)	0.558
Multiple	106 (83.5%)	46 (78.0%)	14 (87.5%)	
Supratentorial\Infratentorial^a^				
Supratentorial	176 (72.1%)	78 (57.8%)	39 (65.0%)	0.017
Infratentorial	68 (27.9%)	57 (42.2%)	21 (35.0%)	
Edema^a^				
Present	193 (79.1%)	109 (80.7%)	46 (76.7%)	0.807
Absent	51 (20.9%)	26 (19.3%)	14 (23.3%)	
Contrast-enhancement^a^				
Heterogeneous	31 (12.7%)	95 (70.4%)	52 (86.7%)	<0.001
Rim-like	207 (84.8%)	38 (28.1%)	8 (13.3%)	
Non-enhancement	6 (2.5%)	2 (1.5%)	0 (0.0%)	
Shape^a^				
Single nodule type (round)	182 (74.6%)	34 (25.2%)	14 (23.3%)	<0.001
Multi-nodular fusion type	47 (19.3%)	82 (60.7%)	41 (68.3%)	
Fuzzy edge type	15 (6.1%)	19 (14.1%)	5 (8.4%)	
Cystic necrosis^a^				
Present	56 (23.0%)	81 (60.0%)	37 (61.7%)	<0.001
Absent	188 (77.0%)	54 (40.0%)	23 (36.2%)	
Low T2WI signal intensity^a^				
Present	15 (6.1%)	3 (2.2%)	26 (43.3%)	<0.001
Absent	229 (93.9%)	132 (97.8%)	34 (56.7%)	

Age, size and volume are shown as mean ± standard deviation; other data are the number of brain metastases with the percentage in parentheses.

a Data are brain metastases for each patient.

### MR image acquisition

MRI images of the brain were acquired using a 3.0 T MRI scanner (Skyra from Siemens Healthineers; Ingenia from Philips Medical Systems, Best, Netherlands). The protocol included the following sequences: non-enhanced fast T1-weighted pulse sequence (T1W), fast T2-weighted pulse sequence with fat suppression (T2W_FS), and T2-weighted axial FLAIR sequence. Enhanced axial T1-weighted pulse sequence and flow-compensated or three-dimensional T1-weighted gradient echo sequence were performed after injection of gadolinium-based contrast agent at a dose of 0.1 millimoles per kilogram adjusted for body weight. Sequence parameters varied between different MRI devices, reflecting the heterogeneity of imaging data in clinical practice.

### Image preprocessing and tumor segmentation

Prior to feature extraction, various preprocessing techniques were applied to improve texture recognition in the images. Firstly, 3D Slicer image processing software^1^ was used to remove all cranial bones from the images and align them to a unified coordinate system. Then, linear image registration tools within 3D Slicer were employed to register each patient’s T1-weighted imaging (T1WI), T2-weighted imaging (T2WI), T2-FLAIR sequence images with contrast-enhanced T1-weighted imaging (CE-T1WI). Subsequently, all registered images were resampled to a pixel size of 1x1x1 mm to ensure consistency in scale and orientation during the extraction of 3D texture features. Additionally, Z-score standardization was applied to normalize each MRI image to zero mean and unit standard deviation using 3D Slicer.

For segmenting intracranial metastatic lesions, 3D Slicer facilitated a semi-automatic contouring algorithm to assist in manually delineating tumor regions. Two radiologists with over 10 years of neuroimaging diagnostic experience outlined tumor regions of interest (ROIs) based on layered review of all images, without knowledge of clinical or pathological details, to ensure accuracy in the final volume of the lesion. The tumor core (encompassing enhanced areas, non-enhanced regions, and possible necrotic tissue) was delineated (13). Tumor boundaries were determined based on enhanced edge on CE-T1WI images. Additionally, cross-verification among T1WI, T2WI, T2-FLAIR, and CE-T1WI was performed to fine-tune tumor contours (14, 15). To ensure reliability in radiomics feature extraction, the same radiologist repeated the segmentation process 2 months later, and intra-observer and inter-observer correlation coefficients (ICC) were calculated. The extraction of radiomic features from ROIs for further analysis was considered only when the ICC coefficient was ≥0.8.

### Feature extraction

Feature extraction was performed using the Radiomics package, an extension plugin in the 3D Slicer software. Extracted features included 18 first-order features, 14 shape features, and 75 texture features (as shown in Table 1). The 75 texture features comprised gray-level co-occurrence matrix (GLCM), gray-level run length matrix (GLRLM), gray-level size zone matrix (GLSZM), and neighborhood gray-tone difference matrix (NGTDM). Additionally, first-order and texture features were calculated based on eight wavelet decompositions (four decompositions of two-dimensional features), resulting in a total of 744 features. Altogether, there were 851 image features. Furthermore, radiomic features were extracted from all registered MRI images, including T1WI, T2WI, T2-FLAIR, and CE-T1WI sequences, resulting in a total of 3,404 image features (851 × 4). For each training dataset, algorithm-based feature selection methods were employed, considering Gini impurity metrics for individual feature selection.

### Model development with machine learning

Before starting the modeling process, 20% of the samples were randomly selected as an independent validation set, while the remaining 80% were used as an independent training set. To address the imbalance issue in the training dataset, Synthetic Minority Over-sampling Technique (SMOTE) was applied to introduce synthetic feature samples and rebalance the minority class data. This data balancing method has been proven to prevent overfitting and enhance model generalization. Data balancing was only applied to the independent training set obtained from the previous random split, while the independent validation set remained unchanged. Z-score standardization was applied to the feature matrix. For each feature vector, the mean and standard deviation were calculated, and then each feature vector was zero-centered and scaled to have unit variance. To avoid selection bias, a Pearson correlation test was conducted on the texture features of BM. Features with a Pearson correlation coefficient exceeding 0.99 were removed to ensure no discrimination among BM from the same patient.

In the feature selection step, a *p*-value-based filtering method was used to rank the features with the strongest discriminative power independently assessing the statistical significance of each feature without analyzing relationships between features. Three feature selection methods—Analysis of Variance (ANOVA), Recursive Feature Elimination (RFE), and Kruskal-Wallis (KW) test—were employed to select features. ANOVA identified significant features related to the labels, RFE selected a subset of features based on the classifier, and KW test excluded the most probable features from the same distribution between two groups. To mitigate overfitting, feature selection was integrated into the model construction process. Specifically, within each group, different feature rankings were obtained using only the training samples. Subsequently, subsets of features were incrementally added from highest to lowest rank. Each feature subset was used to tune model parameters through an internal five-fold cross-validation loop, facilitating model training and evaluation using metrics computed on test samples within the same group (16).

To ensure robustness, three classifiers—Support Vector Machine (SVM), Logistic Regression (LR), and Lasso regularized Logistic Regression (LR-Lasso)—were used to build the model. These machine learning-based classifiers have low tendency for overfitting and are suitable for datasets with numerous heterogeneous predictors and cluster-related observations (e.g., patients with multiple metastases). Linear kernel functions were employed to aid in interpreting the feature parameters of the final model. Additionally, the kernel function mapped features into higher dimensions to find hyperparameters that could differentiate between metastases from different primary sites.

**Figure 2:**
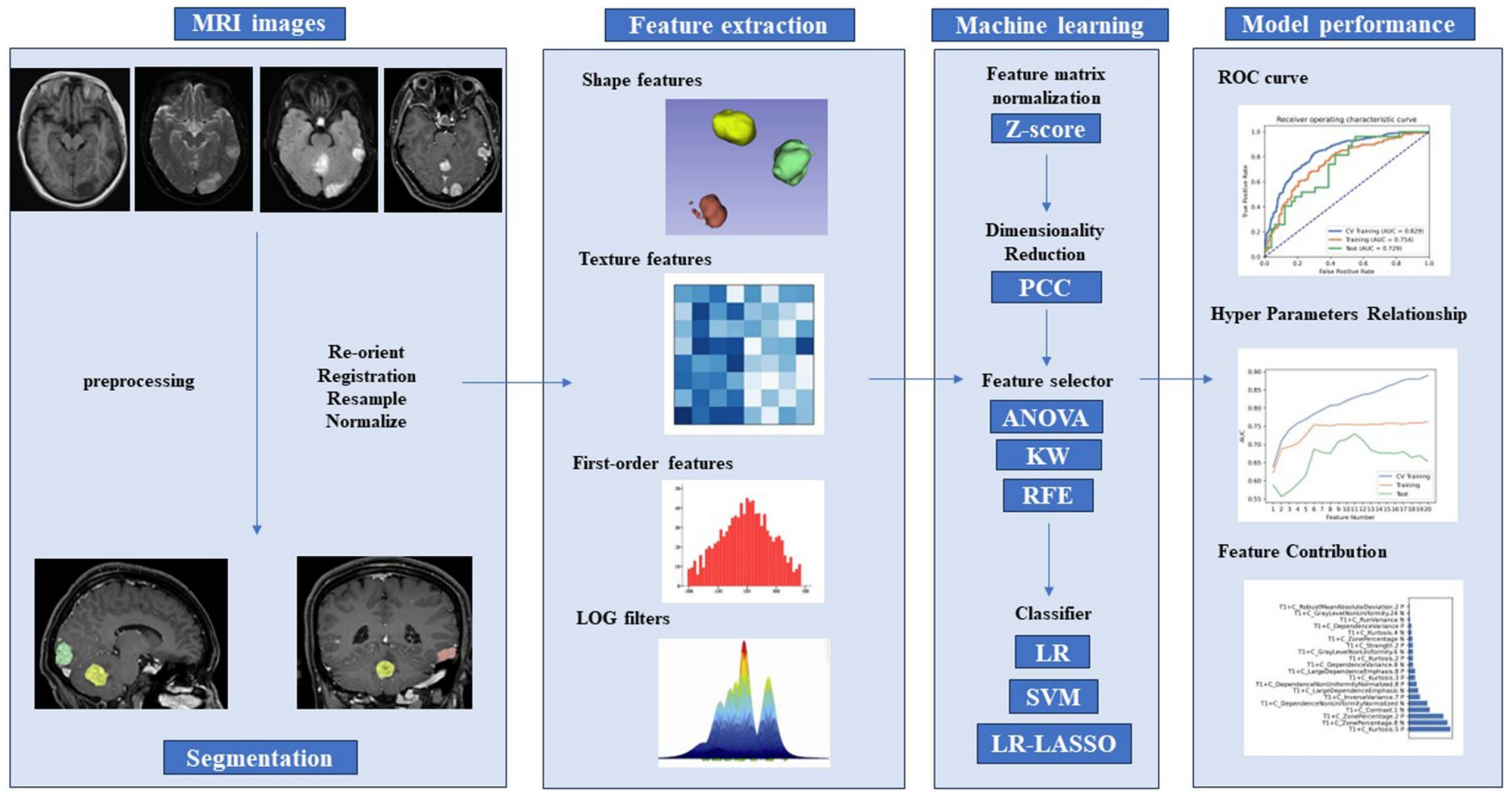
Radiomics pipeline of the study. PCC, Pearson correlation coefficients; ANOVA, analysis of variance; KW, Kruskal–Wallis test; RFE, recursive feature elimination; LR, logistic regression; LASSO, least absolute shrinkage and selection operator; SVM, support vector machine; ROC, receiver operating characteristic curve.

Model validation was conducted based on five-fold cross-validation using independent training and validation sets, which is an external model validation method. Compared to internal cross-validation for model performance estimation, external model validation methods demonstrate stronger robustness for cluster-related data. The Receiver Operating Characteristic (ROC) curve and precision-recall analysis accurately assessed the model’s performance. Performance metrics including sensitivity, specificity, accuracy, area under the curve (AUC), precision-recall area under the curve (PRAUC), positive predictive value (PPV), and negative predictive value (NPV) were computed. Performance metrics (PRAUC and AUC) obtained from the five-fold cross-validation process were compared to select the best model. Feature preprocessing and model exploration were performed using Feature Explorer Pro (FAE) on Python 3.7.6 (V 0.5.8, https://github.com/salan668/FAE). The complete workflow to establish the radiomic model is summarized in Figure 2.

After selecting the optimal radiomic model, clinical variables were incorporated, and the radiomic model was compared with a combined model including both radiomic features and clinical parameters. For each patient, a radiomic score (Rad-score) was generated using a linear combination formula: Rad-score = (value of feature 1 * coefficient of feature 1) + (value of feature 2 * coefficient of feature 2) + … + (value of feature *n* * coefficient of feature n). Clinical parameters were identified through univariate and multivariate logistic analyses.

### Statistical analysis

In this study, statistical analysis was performed using SPSS software (version 24) (17). Mann–Whitney U test and Student’s *t*-test were employed to compare continuous variables, while Pearson’s chi-squared test and Fisher’s exact test were utilized for comparing categorical variables. Logistic regression analysis was conducted to identify significant clinical parameters.

For multi-class scenarios, the area under the Receiver Operating Characteristic curve (AUC) was calculated by assessing the predictive performance of the positive class (i.e., corresponding metastatic tumor type) against all other classes. The ROC curve was generated based on the mean value computed from all cross-validation folds. Given that each machine learning classifier was trained on a unique training dataset and tested using a distinct external validation dataset, the mean AUC serves as an effective estimate of the model’s classification performance in a generalized context. The instability of model predictions (i.e., standard deviation of the ROC curve) was derived from 10 randomly sampled five-fold cross-validation sets. ROC curves and Precision-Recall (PR) curves (utilizing the “precrec” package) were employed for model evaluation, including the calculation of sensitivity and specificity for each diagnostic model. The Youden index is calculated as sensitivity + specificity − 1 (i.e., the Y-axis minus the X-axis of the ROC curve). The maximum value of the Youden index corresponds to the optimal diagnostic cutoff value for the model; the higher its value, the better the diagnostic performance of the model. Performance differences between two models were compared using the DeLong test. Results with a *p*-value below 0.05 were deemed statistically significant.

## Results

### Demographic characteristics of the patient cohorts

Table 1 presents the clinical and MRI characteristics of 202 patients, with a mean age of 60.8 ± 10.3 years, including 87 males and 115 females. Among a total of 439 BMs, the average maximum diameter was 15.7 ± 8.9 mm, with an average volume of 1.8 ± 5.2 cm^3^. Population demographics for the training and validation sets are summarized in Table 2.

**Table 2:** Demographic characteristics of the patients with brain metastases in the training and test sets.

Characteristic	Training cohort (*n* = 160,350)				Test cohort (*n* = 42,89)			
	Lung cancer metastasis	Breast cancer metastasis	Gastrointestinal cancer metastasis	*p* value	Lung cancer metastasis	Breast cancer metastasis	Gastrointestinal cancer metastasis	*p* value
Number of patients, metastasis	101,194	47,108	12,48		26,50	12,27	4,12	
Age (years old)^a^	61.4 ± 10.8	57.1 ± 9.6	61.1 ± 4.6	0.39	60.7 ± 11.2	55.4 ± 8.4	59.7 ± 5.1	0.53
Gender								
Male	57 (56.4%)	4 (8.5%)	8 (66.7%)	<0.001	15 (57.7%)	1 (8.3%)	2 (50%)	0.016
Female	44 (43.6)	43 (91.5%)	4 (33.3%)		11 (42.3%)	11 (91.7%)	2 (50%)	

Data are numbers of brain metastases, with percentages in parentheses.

a Data are means ± standard deviations.

**Figure 3:**
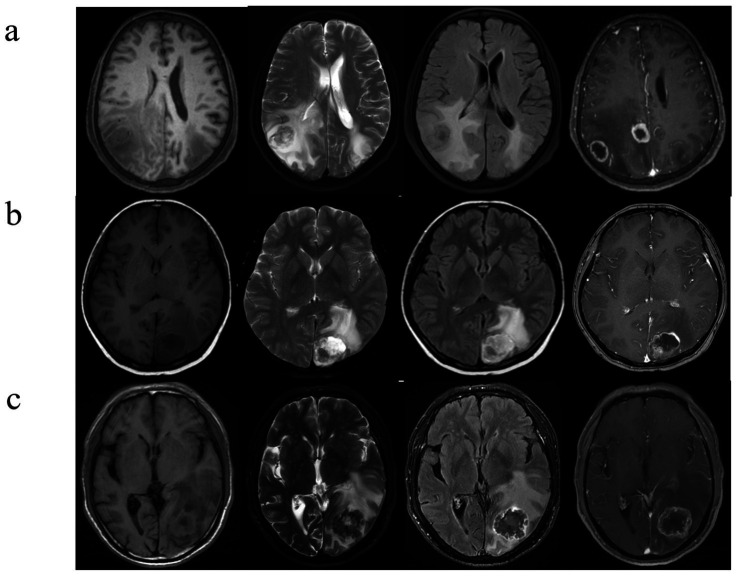
Brain metastases with atypical MRI findings. **(A)** A 73-year-old man with NSCLC. Multiple hyperintense nodules are observed in the bilateral occipital lobes and lateral ventricular trigones on T2WI and FLAIR sequences, accompanied by significant perilesional brain edema, and there is evident ring enhancement on CE- T1WI. **(B)** A 53-year-old woman with a history of breast cancer surgery 3 years ago presents with cystic-solid, lobulated nodules located beneath the cortical layer of the left occipital lobe. The nodules exhibit mixed high signal intensity on both T2WI and FLAIR sequences, along with surrounding peritumoral edema. CE- T1WI reveals significant and heterogeneous enhancement. **(C)** A 42-year-old man, diagnosed with signet ring cell carcinoma on gastroscopy, presents with severe headaches. T2WI shows a low signal intensity nodule in the left occipital lobe, with unclear borders. The lesion demonstrates central hypointensity and peripheral hyperintensity on FLAIR sequences, accompanied by significant peritumoral edema. Additionally, there is heterogeneous ring enhancement surrounding the lesion.

In all three cohorts, six clinical and MR features exhibited statistically significant differences (all *p* < 0.05; Table 1). Among the three cohorts of LC, BC, and GI, the highest proportion of female patients was observed in the BC cohort, with a statistically significant difference (*p* < 0.05). There were no statistically significant differences observed in age distribution, lesion size, or volume (*p* > 0.05). Patients with BM from three distinct primary sites frequently present with multiple intracranial lesions, often accompanied by peritumoral edema (*p* > 0.05). BMLC predominantly localize supratentorially (176 [72.1%]), typically appearing as round, nodular lesions (182 [74.6%]), with enhancement patterns commonly characterized by peripheral rim enhancement (207 [84.8%]) and infrequent cystic degeneration/necrosis (56 [23.0%]). Conversely, BMBC may also occur in the infratentorial compartment (57 [42.2%]), displaying lobulated, multi-nodular fusion morphology (82 [60.7%]), heterogeneous enhancement post-contrast (95 [70.4%]), and more frequent internal necrosis/cystic degeneration (81 [60.0%]). BMGiT may exhibit a typical low signal intensity on T2-weighted imaging (26 [43.3%]) (*p* < 0.05). Figure 3 illustrates representative cases of BM from different primary sites on MRI.

### Diagnostic performance of clinical models

**Figure 4:**
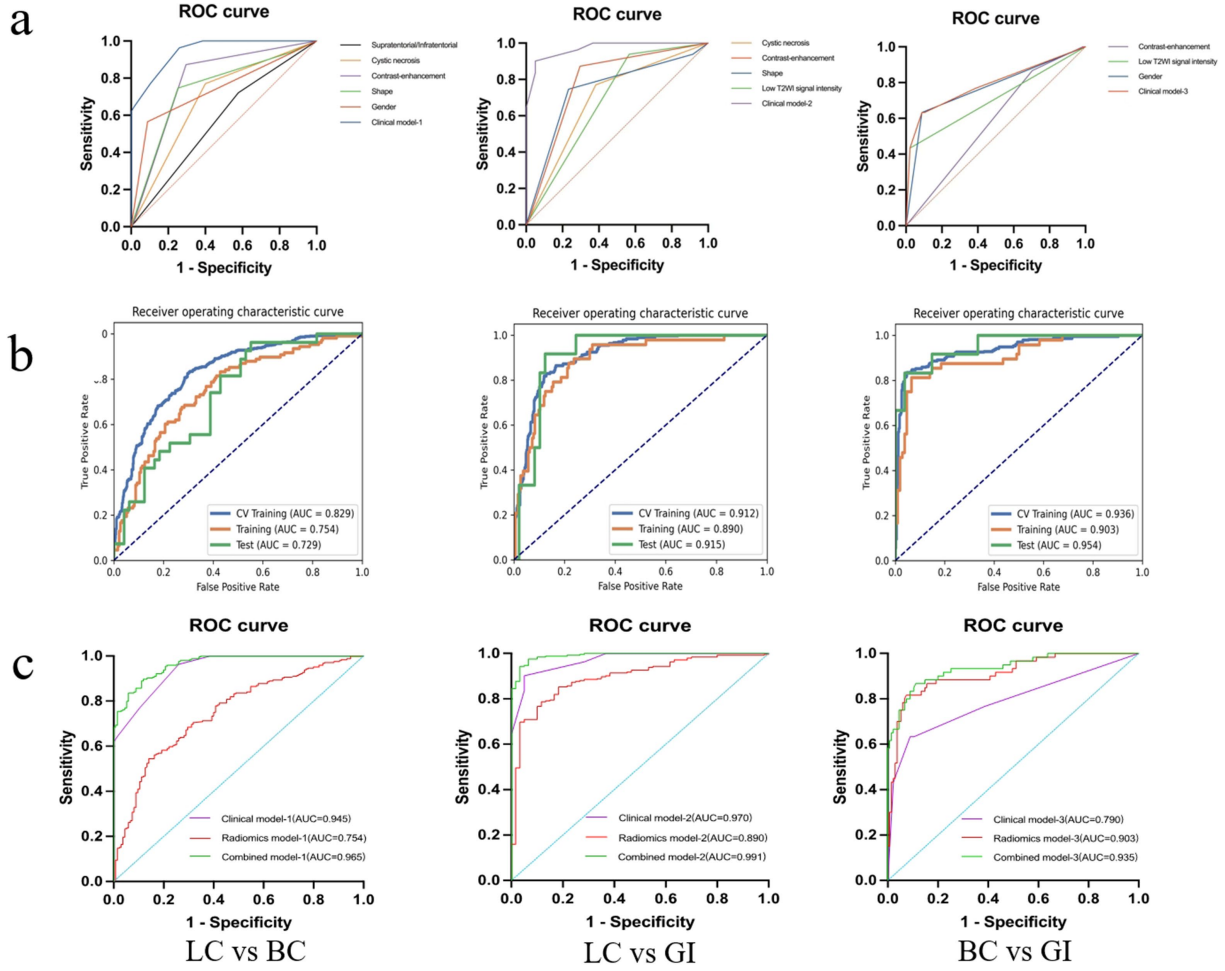
Diagnostic performance of the best performing model. Receiver operator characteristic curves for the clinical, radiomics, and combined model **(A–C)**.

Univariate and multivariate logistic analyses were conducted on basic clinical data (age and sex) and MRI image features of the three cohorts to identify significant clinical parameters. Among these parameters, features such as sex and presence of T2WI low signal were found to be independent predictors. The diagnostic performance AUC ranged from 0.790 (corresponding to BMBC vs. BMGiT) to 0.970 (corresponding to BMLC vs. BMGiT) across the three models (Figure 4). Comparative analysis on randomly sampled five-fold cross-validation sets revealed low variability in results, indicating stable predictive performance.

### Diagnostic performance of radiomics models

**Figure 5:**
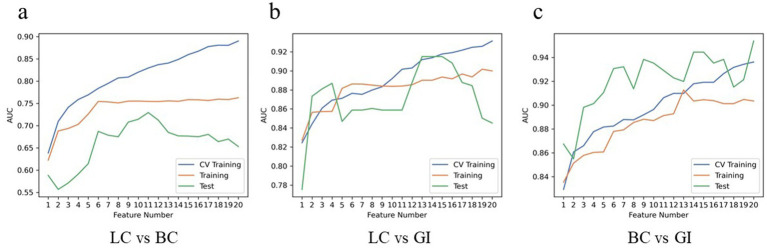
Hyper parameters relationship of the training cohort, cross-validation cohort, and test cohort. The curve illustrates the correlation between the number of features and the model’s AUC value, aiding in the determination of the optimal number of features for the model. In LC vs. BC of the test cohort, the model attains a peak AUC value of 0.729 with 11 features **(A)**; In LC vs. GI of the test cohort, the model attains a peak AUC value of 0.915 with 13 features **(B)**; In BC vs. GI of the test cohort, the model attains a peak AUC value of 0.954 with 20 features **(C)**. LC, lung cancer; BC, breast cancer; GI, gastrointestinal (cancer).

The diagnostic performance of radiomics models for BM in the three cohorts is summarized in Table 3. In the classification models of BMLC versus BMBC, the highest AUC (0.829) and PRAUC (0.818) were achieved in the five-fold cross-validation set. At this point, the model achieved AUC and PRAUC of 0.754 and 0.616, respectively, on the training set, and 0.729 and 0.578, respectively, on the independent validation set. This process utilized RFE feature selector and SVM classifier, selecting 11 features for the model (Figure 5).

**Table 3:** The diagnostic performance of radiomics models for all patients.

	AUC (95% CI)	PRAUC	Accuracy	Sensitivity	Specificity	PPV	NPV	Youden Index
Lung cancer vs. Breast cancer								
Cross-validation	0.829 (0.809–0.850)	0.818	0.764	0.825	0.704	0.736	0.801	0.528
Training cohort	0.754 (0.698–0.811)	0.616	0.672	0.787	0.608	0.528	0.837	0.395
Test cohort	0.729 (0.616–0.843)	0.578	0.618	0.852	0.490	0.479	0.857	0.342
Lung cancer vs. Gastrointestinal cancer								
Cross-validation	0.912 (0.898–0.926)	0.896	0.851	0.830	0.872	0.867	0.837	0.702
Training cohort	0.890 (0.841–0.940)	0.641	0.802	0.875	0.784	0.500	0.962	0.659
Test cohort	0.915 (0.844–0.986)	0.583	0.836	0.917	0.816	0.55	0.976	0.733
Breast cancer vs. Gastrointestinal cancer								
Cross-validation	0.936 (0.920–0.953)	0.939	0.895	0.836	0.954	0.948	0.853	0.789
Training cohort	0.903 (0.848–0.958)	0.836	0.897	0.813	0.935	0.848	0.918	0.748
Test cohort	0.954 (0.889–1.000)	0.924	0.923	0.833	0.963	0.909	0.929	0.796

AUC, area under the curve; ACC, accuracy; CI, confidence interval; NPV, negative predictive value; PRAUC, area under the precision-recall curve; PPV, positive predictive value; Sen, sensitivity; Spe, specificity.

In the classification models of BMLC versus BMGiT, the process employing ANOVA feature selector and SVM classifier obtained the highest AUC (0.912) and PRAUC (0.896) in the five-fold cross-validation set. Thirteen features were selected for the model (Figure 5). At this point, the model achieved AUC and PRAUC of 0.890 and 0.641, respectively, on the training set, and 0.915 and 0.583, respectively, on the independent validation set.

In the classification models of BMBC versus BMGiT, the process utilizing KW feature selector and SVM classifier obtained the highest AUC (0.936) and PRAUC (0.939) in the five-fold cross-validation set. Twenty features were selected for the model (Figure 5). At this point, the model achieved AUC and PRAUC of 0.903 and 0.836, respectively, on the training set, and 0.954 and 0.924, respectively, on the independent validation set.

In summary, Figure 4 presents the correlation curve between the number of model features and AUC values, used to determine the optimal number of model features for diagnostic performance. One-on-one analysis demonstrates that using a small number of features (13 and 20 features, respectively) from the optimal dataset can accurately differentiate BMGiT from BMLC (AUC = 0.915 ± 0.071) and BMBC (AUC = 0.954 ± 0.064). However, accuracy is not desirable when distinguishing BMLC from BMBC (AUC = 0.729 ± 0.114), indicating that these features are not suitable for classifying these types of BM.

### Diagnostic performance of combined models

**Figure 6:**
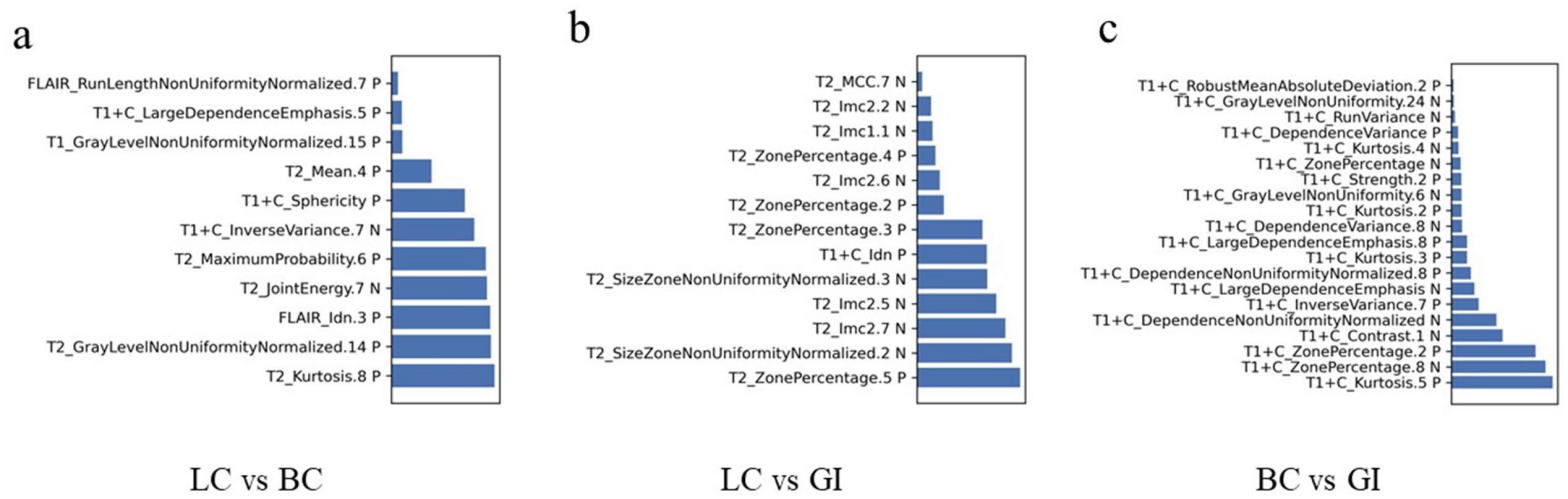
Radiomic feature distributions between metastatic tumor types. Bar chart shows normalized means of the most important features that were jointly selected during all training procedures. **(A)** In the model of LC vs. BC, the T2_Kurtosis.8 texture parameter makes the highest degree of contribution, with the coefficient of 0.569; **(B)** In the model of LC vs. GI, the T2_ZonePercentage.5 texture parameter makes the highest degree of contribution, with the coefficient of 1.112; **(C)** In the model of BC vs. GI, the T1 + C_Kurtosis.5 texture parameter makes the highest degree of contribution, with the coefficient of 1.651. C, contrast enhanced; T1, T1 weighted; T2, T1 weighted.

Using bar chart analysis, the contribution differences of radiomics features in different BM classification models were determined (Table 4; Figure 6). Results indicate that in the BMLC versus BMBC classification model, the T2_Kurtosis.8 texture parameter had the highest contribution with a coefficient of 0.569. In the BMLC versus BMGiT classification model, the T2_ZonePercentage.5 texture parameter had the highest contribution with a coefficient of 1.112. In the BMBC versus BMGiT classification model, the T1 + C_Kurtosis.5 texture parameter had the highest contribution with a coefficient of 1.651.

**Table 4:** Top features of the best dataset (3D features) ranked according to their coefficients in the model of one-versus-one analysis.

Lung cancer vs. Breast cancer		Lung cancer vs. Gastrointestinal cancer		Breast cancer vs. Gastrointestinal cancer	
Feature	Coef in model	Feature	Coef in model	Feature	Coef in model
FLAIR_Idn.3	0.545	T1 + C_Idn	0.755	T1 + C_Contrast.1	−0.834
FLAIR_RunLengthNonUniformityNormalized.7	0.037	T2_Imc1.1	−0.169	T1 + C_DependenceNonUniformityNormalized	−0.734
T1 + C_InverseVariance.7	−0.456	T2_Imc2.2	−0.153	T1 + C_DependenceNonUniformityNormalized.8	0.320
T1 + C_LargeDependenceEmphasis.5	0.058	T2_Imc2.5	−0.855	T1 + C_DependenceVariance	0.112
T1 + C_Sphericity	0.405	T2_Imc2.6	−0.245	T1 + C_DependenceVariance.8	−0.173
T1_GrayLevelNonUniformityNormalized.15	0.060	T2_Imc2.7	−0.954	T1 + C_GrayLevelNonUniformity.24	−0.043
T2_GrayLevelNonUniformityNormalized.14	0.549	T2_MCC.7	−0.057	T1 + C_GrayLevelNonUniformity.6	−0.169
T2_JointEnergy.7	−0.528	T2_SizeZoneNonUniformityNormalized.2	−1.024	T1 + C_InverseVariance.7	0.445
T2_Kurtosis.8	0.569	T2_SizeZoneNonUniformityNormalized.3	−0.757	T1 + C_Kurtosis.2	0.172
T2_MaximumProbability.6	0.521	T2_ZonePercentage.2	0.290	T1 + C_Kurtosis.3	0.259
T2_Mean.4	0.22	T2_ZonePercentage.3	0.706	T1 + C_Kurtosis.4	−0.116
		T2_ZonePercentage.4	0.199	T1 + C_Kurtosis.5	1.651
		T2_ZonePercentage.5	1.112	T1 + C_LargeDependenceEmphasis	−0.377
				T1 + C_LargeDependenceEmphasis.8	0.259
				T1 + C_RobustMeanAbsoluteDeviation.2	0.038
				T1 + C_RunVariance	−0.059
				T1 + C_Strength.2	0.164
				T1 + C_ZonePercentage	−0.152
				T1 + C_ZonePercentage.2	1.372
				T1 + C_ZonePercentage.8	−1.534

FLAIR, fluid-attenuated inversion recovery; T1, T1-weighted; T2, T2-weighted; T1 + C, contrast-enhanced T1-weighted.

Based on the AUC and PRAUC of all radiomics models, a Rad-score was calculated using the integrated radiomics model. The comprehensive model combines independent clinical parameters and Rad-score using logistic regression. The AUC values of the combined model were 0.965 for BMLC versus BMBC, 0.991 for BMLC versus BMGiT, and 0.935 for BMBC versus BMGiT (Figure 4).

## Discussion

Typical MRI imaging features suggest that BM from LC often appear multiple, round or oval-shaped, scattered in the subcortical white matter. Even when the lesions are <1 cm, peritumoral vasogenic edema is still prominent, presenting as ring enhancement upon contrast administration. BM from BC frequently occur as solitary or multiple lesions, showing a lobulated or cystic-solid mixed composition, with notable necrosis and cystic changes. Post-contrast enhancement reveals uneven enhancement, often associated with leptomeningeal metastases. BMGiT may manifest as lesions containing mucinous or protein-rich components, displaying low signal intensity on T2-weighted imaging (18, 19). Our study also incorporated the analysis of various clinical indicators and imaging features, yielding results consistent with existing literature. However, distinguishing BM from different primary sites solely based on these MRI imaging features is highly challenging, especially when tumors present as single enhanced lesions surrounded by edema or exhibit atypical imaging characteristics.

When conducting analysis solely using MRI image features, the highest AUC value (0.970) was achieved for BM from LC and GI, while BM from BC and GI yielded the lowest AUC value (0.790) (Figure 4). This discrepancy reflects the less distinct expression of specific features for BC and GI, possibly due to the extensive spectrum of clinical features and greater tissue heterogeneity, making the interpretation of imaging biomarkers for these BM complex. Some research findings (9, 20) support our observations, exploring clustering analysis of image features for non-small cell lung cancer subtypes. It was noted that when employing classifier models based solely on image features, the AUC values were relatively lower, suggesting inadequate differentiation of features for these tumor types within the underlying image feature set.

“Radiomics” is an emerging medical imaging analysis method that transforms images into quantitative data, thereby improving the accuracy of diagnosis, prognosis assessment, and treatment response evaluation, ultimately aiding in better clinical decision-making. We modeled the 3D texture features of MRI images, and the results showed that in three models encompassing most of the major tumor types causing BM, radiomics analysis of 3D texture features could differentiate BMLC from BMBC and BMGiT. The average AUC values for BMLC vs. BMBC, BMBC vs. BMGiT, and BMLC vs. BMGiT were 0.729, 0.954, and 0.915, respectively.

Our study does not represent the first attempt to utilize texture features to distinguish the primary cancer sites of BM. In the study by Moratal et al. (21), the discriminatory power of 2D and 3D MRI texture features was compared, and various classifiers were tested to classify BM from LC and melanoma. Beres et al. (22) investigated the statistical significance of 2D and 3D texture features extracted from histograms and GLCM to differentiate BM of LC and BC. Our work enhances this research by exploring additional texture features (including patients with GI) and considering machine learning approaches. Based on our findings, we support the conclusion of Beres et al. that texture analysis may aid in distinguishing BM originating from different primary tumors.

We attempted to interpret the results of our model by analyzing the selection frequency of radiomic features and applying SHA*p* value analysis, which assigns importance values to each feature for specific predictions (23). In this study, the best-performing classifier was the Support Vector Machine (SVM) classifier, a machine learning-based algorithm known for its high robustness and low tendency for overfitting, suitable for datasets with numerous heterogeneous predictive factors and cluster-related observations. According to the SHAP value analysis of the best-performing model, the most contributory features for multiclass BM classification were “T2_Kurtosis.8,” “T2_ZonePercentage.5,” and “T1 + C_Kurtosis.5” (Table 4).

The feature “Kurtosis.8” from T2WI was particularly useful in distinguishing between BMLC and BMBC, showing that higher values led to higher decision scores attributed to BMBC, while lower values led to attribution to BMLC. Kurtosis is a statistical measure describing the sharpness of the shape of a local intensity distribution, quantifying the thickness of the tails of the data distribution and the degree to which the data is centered, with larger values associated with increased differences in intensity values between adjacent voxels (24). Therefore, based on our research findings, it seems that more uneven and higher signal intensity on T2WI favors the diagnosis of BMBC.

On the other hand, the feature “ZonePercentage.5” from T2WI was useful in differentiating between BMLC and BMGiT, with lower and higher values, respectively, associated with BMGiT and BMLC. This may reflect the typical T2WI hypointensity of BMGiT due to their high cell density (25, 26).

When distinguishing between BMBC and BMGiT, the feature “Kurtosis.5” from T1-CE seemed to play an important role, with higher values indicating BMBC over BMGiT. Kurtosis, a statistical measure describing the sharpness of the shape of a probability distribution, with positive kurtosis distributions sharper and thicker-tailed than the normal distribution; whereas negative kurtosis distributions flatter and sparser-tailed than the normal distribution (24). The contribution coefficient of “Kurtosis.5” from T1-CE reached 1.651, the highest among all selected texture features, possibly indicating that the enhancement degree of BMBC on T1-CE is more significant and uneven compared to BMGiT.

From our results, it is evident that most features originated from first-order histogram features (kurtosis, mean, mean absolute deviation) of T2WI and T1-CE images, indicating significant differences in cell density and distribution among different tumor types (27, 28). Radiologically, BMBC often exhibit necrosis, cystic changes, significant enhancement post-contrast, and heterogeneous enhancement, while BMGiT may present with typical hypointensity on T2WI images, which could be the physiological basis for selecting these histogram features. Other texture features like GLCM, GLRLM, and GLDM also reflect the heterogeneity within tumors (27).

Radiomics provides information that cannot be revealed through visual inspection and can offer indirect reference to potential biological differences at the tissue and cellular levels (29). However, further research is needed to explain the relationship between predictive factors based on radiomics models and outcomes, such as the SHAP value analysis used in this study, to address the “black box” issue of current radiomics models and enhance the role of this method in clinical decision-making.

Our study has several limitations that need to be addressed. Firstly, the small sample size due to the lack of standardized multicenter data hinders the generalizability and stability of our results. Enlarging the sample size is crucial to enhance these aspects. Secondly, our study only utilized routine MRI sequences (T1WI, T2WI, T2-FLAIR, T1-CE) for radiomic analysis. Including additional MRI sequences like DWI and perfusion imaging in future studies can help capture the differences among BM from different primary sites. Excluding low-resolution images in our study was necessary to ensure meaningful texture analysis of small lesions. However, including these images might decrease predictive performance. Deep learning preprocessing of poor-quality images could be a potential solution, but further research is required in this area (30). Thirdly, the semi-automatic tumor ROI segmentation in our study implies some level of observer dependency in the machine learning process. To mitigate this, we employed consensus ROIs. Furthermore, previous studies have indicated the considerable stability of radiomic features in segmentation variations (31).

Addressing these limitations, we are encouraged by initiatives such as the Imaging Biomarker Standardization Initiative and the Quantitative Imaging Network established by the National Institutes of Health (32, 33). Future research endeavors should leverage standardized high-resolution images, incorporate imaging sequences capturing additional tissue features, and integrate comprehensive clinical data to explore the full potential of radiomics-based diagnostics.

## Conclusion

Machine learning-based radiomics models exhibit a high degree of accuracy in distinguishing BM originating from various primary sites. Leveraging the proposed tumor type prediction model as an adjunctive decision support tool has the potential to streamline diagnostic workflows and expedite the localization of primary lesions.

## Data Availability

The original contributions presented in the study are included in the article/supplementary material, further inquiries can be directed to the corresponding author.

## Footnotes

^1^ https://www.slicer.org/
